# Fatty acid 16:4(n-3) stimulates a GPR120-induced signaling cascade in splenic macrophages to promote chemotherapy resistance

**DOI:** 10.1096/fj.201601248R

**Published:** 2017-02-09

**Authors:** Julia M. Houthuijzen, Ilse Oosterom, Brian D. Hudson, Akira Hirasawa, Laura G. M. Daenen, Chelsea M. McLean, Steffen V. F. Hansen, Marijn T. M. van Jaarsveld, Daniel S. Peeper, Sahar Jafari Sadatmand, Jeanine M. L. Roodhart, Chris H. A. van de Lest, Trond Ulven, Kenji Ishihara, Graeme Milligan, Emile E. Voest

**Affiliations:** * Department of Molecular Oncology, Netherlands Cancer Institute, Amsterdam, The Netherlands;; † Department of Molecular Pathology, Netherlands Cancer Institute, Amsterdam, The Netherlands;; ‡ Department of Medical Oncology, University Medical Center Utrecht, Utrecht, The Netherlands;; § Centre for Translational Pharmacology, Institute of Molecular, Cell, and Systems Biology, University of Glasgow, Glasgow, United Kingdom;; ¶ Department of Genomic Drug Discovery Science, Kyoto University, Kyoto, Japan;; ‖ Department of Physics, Chemistry, and Pharmacy, University of Southern Denmark, Odense, Denmark;; # Department of Biochemistry and Cell Biology, Faculty of Veterinary Medicine, Utrecht University, Utrecht, The Netherlands;; ** National Research Institute of Fisheries Science, Kanazawaku, Japan

**Keywords:** FFAR4, GPR40, FFAR1, PIFA

## Abstract

Although chemotherapy is designed to eradicate tumor cells, it also has significant effects on normal tissues. The platinum-induced fatty acid 16:4(n-3) (hexadeca-4,7,10,13-tetraenoic acid) induces systemic resistance to a broad range of DNA-damaging chemotherapeutics. We show that 16:4(n-3) exerts its effect by activating splenic F4/80^+^/CD11b^low^ macrophages, which results in production of chemoprotective lysophosphatidylcholines (LPCs). Pharmacologic studies, together with analysis of expression patterns, identified GPR120 on F4/80^+^/CD11b^low^ macrophages as the relevant receptor for 16:4(n-3). Studies that used splenocytes from GPR120-deficient mice have confirmed this conclusion. Activation of the 16:4(n-3)-GPR120 axis led to enhanced cPLA_2_ activity in these splenic macrophages and secretion of the resistance-inducing lipid mediator, lysophosphatidylcholine(24:1). These studies identify a novel and unexpected function for GPR120 and suggest that antagonists of this receptor might be effective agents to limit development of chemotherapy resistance.—Houthuijzen, J. M., Oosterom, I., Hudson, B. D., Hirasawa, A., Daenen, L. G. M., McLean, C. M., Hansen, S. V. F., van Jaarsveld, M. T. M., Peeper, D. S., Jafari Sadatmand, S., Roodhart, J. M. L., van de Lest, C. H. A., Ulven, T., Ishihara, K., Milligan, G., Voest, E. E. Fatty acid 16:4(n-3) stimulates a GPR120-induced signaling cascade in splenic macrophages to promote chemotherapy resistance.

Since their deorphanization as GPCRs that respond to a diverse range of both saturated and unsaturated long-chain fatty acids, there has been growing interest in both GPR40 (systematically named free fatty acid receptor 1) and GPR120 (free fatty acid receptor 4) (1, 2). Both receptors are expressed by a number of endocrine cells, including cells of the pancreas and entero-endocrine cells of the gut (3–5). Here, free fatty acid–induced release of insulin, incretins, and satiety-regulating hormones have focused attention on the potential of synthetic agonists of one or both of these receptors to provide novel approaches to regulate glycaemia and to treat metabolic disorders, such as type II diabetes (4, 6–8); however, as with most other GPCRs, both GPR40 and GPR120 are thought to be expressed more broadly. This includes immune cell subsets (9, 10), lung (11–13), and precursors of bone and adipocytes (9, 13–15), and there is evolving interest in whether this might translate into opportunities for ligands that target these receptors in wider therapeutic settings.

Although both GPR40 and GPR120 can be activated by a range of long-chain free fatty acids (4), including n-3 fatty acids that are routinely considered to be health beneficial (16, 17), more attention has been directed toward n-3 fatty acids as activators of GPR120 (4, 9). Furthermore, quantitatively minor lipids, including branched palmitic acid esters of hydroxy stearic acids, have more recently been suggested to produce key effects as activators of GPR120 (18).

There has been growing evidence that fatty acid–induced activation of GPR40 and/or GPR120 can regulate tumor growth and migration of various cancer types, including melanoma and prostate cancers (19, 20). We have previously identified mechanisms of reversible and systemic chemotherapy resistance that involve lipid-mediated crosstalk between mesenchymal stem cells, splenic macrophages, and tumor cells. Within these studies, we showed that mesenchymal stem cells can secrete 2 distinct polyunsaturated fatty acids {12-*S*-hydroxy-5,8,10-heptadecatrienoic acid (12-S-HHT) and hexadeca-4,7,10,13-tetraenoic acid [16:4(n-3)]} upon stimulation with platinum-containing chemotherapeutics (Supplemental Fig. 1) (21). These platinum-induced fatty acids (PIFAs) convey resistance to a broad range of DNA-damaging chemotherapeutics in various tumor mouse models (21). PIFAs do not induce resistance directly to tumor cells but rather function *via* F4/80^+^/CD11b^low^ macrophages that are located in the red pulp of the spleen (22).

Herein, we assess the contribution of free fatty acid receptors GPR40 and GPR120 to chemoresistance induced by 16:4(n-3), as we show that these GPCRs are expressed exclusively by the F4/80^+^/CD11b^low^ subpopulation of splenic macrophages that are known to induce chemoresistance. By using combinations of selective pharmacologic activation and inhibition of GPR40 and GPR120 in concert with splenocytes isolated from both wild-type and GPR120-deficient mice, we show that effects of 16:4(n-3) are induced specifically *via* GPR120 and that activation of this receptor results in a signaling cascade within splenocytes that involves cytosolic PLA_2_-mediated generation and release of a specific isoform of lysophosphatidylcholine (LPC), which acts as the ultimate inducer of chemoresistance.

## MATERIALS AND METHODS

### Reagents

16:4(n-3) was isolated from *Ulva pertusa* as previously described (23). GW1100 was purchased from Cayman Chemical (Ann Arbor, MI, USA). GW9508 and AACOCF_3_ (arachidonyl trifluoromethyl ketone) were purchased from Tocris (Bristol, United Kingdom). NCG21, TUG-891, AH-7614, and TUG-1197 were synthesized as described previously (24–27). For fluorescence-activated cell sorting (FACS) analysis, the following Abs were used: rat anti-mouse F4/80-FITC and rat anti-mouse CD11b-APC (both from eBioscience, San Diego, CA, USA). For immunohistochemical staining, the following Abs were used: anti-γH2AX (gamma histone 2A family member X2577; Cell Signaling Technologies, Danvers, MA, USA), anti-GPR120 (NBP1-00858; Novus Biologicals, Littleton, CO, USA) and poly–horseradish peroxidase (HRP) goat anti-rabbit/rat/mouse (Immunologic, Duiven, The Netherlands). For Western blotting, the following Abs were used: rabbit anti–phospho-cPLA_2_ (Ser505, 2831; Cell Signaling Technologies), mouse anti–β-actin (NB600-501; Novus Biologicals), and goat anti-mouse HRP (Santa Cruz Biotechnology, Santa Cruz, CA, USA).

### Animal models

C26 cells were implanted in BALB/c and LLC cells were implanted in C57bl/6 mice (both from Charles River Labs, Northampton, MA, USA). For all experiments, 8- to 10-wk-old male mice were used. At d 0, mice were subcutaneaously injected with 1 × 10^6^ (for C26) or 0.5 × 10^6^ (for LLC) tumor cells. Mice were splenectomized 1 d after tumor cell injection. Spleens from the surgery were used to prepare splenic conditioned medium (sCM). At d 8 (C26) or d 10 (LLC), when the tumors reached a size of 50–100 mm^3^, animals were randomly assigned to groups and treatment was started. Mice received an intraperitoneal injection of 6 mg/kg cisplatin alone or in combination with an subcutaneaous injection of 200 µl sCM or 100 μl of LPC(24:1) or LPC(24:0), both 10 nmol. Blinded tumor volume measurements were taken once every 2 d by using a digital caliper. Tumor volume was determined as length × width^2^ × 0.5. Control mice received appropriate vehicles. All experimental animal procedures conducted in Utrecht, The Netherlands, were approved by the University Medical Center Animal Ethics Committee and were in agreement with the current Dutch laws on animal experiments. All experimental animal procedures conducted in Kyoto, Japan, were approved by the Kyoto University Animal Care and Use Committee. To show a difference of 20% in tumor volume with an sd of 10% and a type I error (α) of 5% using a power of 90%, a minimum of 8 mice per treatment group were required.

### Cell lines

C26 and LLC cells (both from American Type Culture Collection, Manassas, VA, USA) were grown in DMEM (4.5 g glucose/L) + 5% fetal calf serum at 5% CO_2_ and 37°C. Flp-In T-REx 293 cells with inducible overexpression of GPR120-enhanced yellow fluorescent protein (eYFP) or GPR40-eYFP were grown in DMEM (4.5 g glucose/L) + 10% fetal calf serum at 5% CO_2_ and 37°C. All cell lines were mycoplasma negative, and C26 and LLC cells were also tested for mouse pathogens according to the Federation of European Laboratory Animal Science Associations panel and were found to be negative.

### Preparation of sCM from splenocytes

Spleens from splenectomized mice were used to make sCM. Single-cell suspensions were made from spleens and red blood cells were lysed by using red blood cell lysis buffer (155 mM NH_4_Cl, 10 mM KHCO_3_, and 0.1 mM EDTA). Splenocytes were sorted by magnetic bead sort to isolate F4/80^+^ macrophages before 16:4(n-3) and 12-S-HHT incubation according to a previously described protocol (22). Magnetic-activated cell sorted (MACS)-isolated splenocytes were incubated in serum-free medium (DMEM, 4.5 g glucose/L) with 25 nM 16:4(n-3) or 20 nM 12-S-HHT for 1 h at 5% CO_2_ and 37°C. After incubation, supernatant from the splenocytes was harvested, filtered through a 0.2-μm filter, and stored at −80°C until use. For preparation of sCM with AH-7614, splenocytes were preincubated for 10 min with 1 μM AH-7614 before adding 16:4(n-3) for an additional 1 h. Splenocytes that were incubated with 16:4(n-3) and GW1100 or AACOCF_3_ were preincubated for 30 min with 10 μM of the compounds before addition of 16:4(n-3). GW9508 (100 μM), NCG21 (100 μM), TUG-891 (10 μM), and TUG-1197 (10 μM) were incubated with splenocytes for 1 h before harvesting sCM as described above.

### FACS sorting and quantitative PCR analysis of GPCRs

Single-cell suspensions were made from the spleen, and red blood cells were lysed as described above. Next, splenocytes were incubated with F4/80-FITC and CD11b-APC (concentration 1 μl/1 × 10^7^ cells/100 μl) or corresponding isotype controls (FITC and APC; both from eBioscience) in FACS buffer for 30 min on ice. F4/80^+^/CD11b^low^, F4/80^+^/CD11b^high^, F4/80^−^/CD11b^high^ and F4/80^−^/CD11b^−^ populations were sorted by using a FACSAria III (BD Bioscience, Brea, CA, USA). Gates for sorting were set according to unstained and isotype control–stained samples. RNA was extracted from FACS-sorted splenic populations by using Trizol (Thermo Fisher Scientific, Waltham, MA, USA). cDNA was synthesized by using Superscript II (Thermo Fisher Scientific) according to manufacturer protocol using oligo dTs (Thermo Fisher Scientific). Mouse primers used were as follows: for detection of GPR40, forward: 5′-GCTATTCCTGGGGTGTGTGT-3′, reverse: 5′-CCCTGTGATGAGTCCTAACT-3′; GPR41, forward: 5′-CTGCTCCTGCTCCTCTTC-3′, reverse: 5′-CCAGGCGACTGTAGCAGTA-3′; GPR43, forward: 5′-GGCTTCTACAGCAGCATCTA-3′, reverse: 5′-AAGCACACCAGGAAATTAAG-3′; GPR84, forward: 5′-TCCAATTCTGTCTCCATCCT-3′, reverse: 5′-CTGACTGGCTCAGATGAAA-3′; and GPR120, forward: 5′-CCATCCCTCTAGTGCTCGTC-3′, reverse: 5′-TGCGGAAGAGTCGGTAGTCT-3′. For quantitative PCR analysis of human spleen, CD163^+^ and CD163^−^ splenocytes were first isolated by magnetic bead sort using the same protocol as for mouse spleen except for the Abs (22). Abs used were as follows: mouse anti-human CD163-APC (concentration: 5 μl/1 × 10^7^ cells/100 μl; eBioscience) and anti-APC (concentration: 10 μl/1 × 10^7^ cells/100 μl; Miltenyi Biotec, Bergisch Gladbach, Germany). Next, RNA was also isolated by using Trizol, and cDNA was synthesized by using Superscript II. Primers used for detection of human GPCRs were: GPR40, forward: 5′-GTGTCACCTGGGTCTGGTCT-3′, reverse: 5′-GAGCAGGAGAGAGAGGCTGA -3′; GPR41, forward: 5′-TCTCAGCACCCTGAACTCCT -3′, reverse: 5′-TTCTGCTCCTTCAGCTCCAT-3′; GPR43, forward: 5′-AAGGAGAAGGGATGCCAAGT-3′, reverse: 5′-GGGATACCAAGCTGGTGAAA -3′; GPR84, forward: 5′-TCAGCAGTGTTGGCATCTTC -3′, reverse: 5′-TAACCTGCTGTCCAGCTCCT-3′; GPR120, forward: 5′-CTTCTTCTCCGACGTCAAGG-3′, reverse: 5′-AGAGGGATAGCGCTGATGAA-3′; and GAPDH (glyceraldehyde 3-phosphate dehydrogenase), forward: 5′- CGACCACTTTGTCAAGCTCA-3′, reverse: 5′- AGGGGTCTACATGGCAACTG-3′.

### Immunohistochemistry

For immunohistochemistry experiments, murine tumors were harvested 4 h after therapy. Healthy human spleen tissue was provided from the pathology biobank database of the Netherlands Cancer Institute. Formaldehyde-fixed, paraffin-embedded tissue sections were deparaffinated, rehydrated, and incubated in endogenous peroxidase blocking buffer that contained 5% H_2_O_2_. Antigen retrieval was performed by cooking slides in citrate buffer. Tissue slides were blocked in 5% goat serum (Thermo Fisher Scientific Life Sciences) in Tris-buffered saline/Tween 20 (TBST) for γH2AX staining or in serum-free protein block (Dako, Carpinteria, CA, USA) for GPR120 staining. Abs were used in the following dilutions: rabbit anti-γH2AX (1:200) in 5% goat serum in TBST overnight at 4°C (Cell Signaling Technologies) and rabbit anti-GPR120 (1:1000) in Bright Diluent (Immunologic), also overnight at 4°C. Poly-HRP goat anti-rabbit/rat/mouse (Immunologic) was used as secondary Ab, followed by 3,3′-diaminobenzidine staining. Slides were counterstained with hematoxylin. Each tumor slide was scanned by using a Leica Aperio Scanscope (Leica, Wetzlar, Germany), and a minimum of 10 images per tumor were analyzed. To determine the percentage of positive cells, a grid with 54 intersection points was used. A second examiner who was blinded to the treatment groups also quantified γH2AX staining.

### Western blot

F4/80^+^ cells were isolated from murine spleens by using magnetic bead sorting. Spleens were harvested from non–tumor-bearing mice and a single-cell suspension was made. After red blood cell lysis, splenocytes were resuspended in FACS buffer [1% bovine serum albumin (BSA), 5 mM EDTA in PBS, pH 7.4] and counted. Splenocytes were incubated with rat anti-mouse F4/80 at a concentration of 3 μl/1 × 10^7^ cells in 100 μl for 30 min on ice. After incubation, cells were washed in FACS buffer and incubated with goat anti-rat microbeads at a concentration of 5 μl/1 × 10^7^ cells in 100 μl for 15 min on ice (22). Again, cells were washed after Ab incubation, resuspended in FACS buffer, and separated according to manufacturer instructions using LS columns (Miltenyi Biotec). F4/80^+^ splenocytes were incubated for indicated times with 25 nM 16:4(n-3). After incubation, cells were lysed and subjected to SDS-PAGE and Western blotting. Membranes were blocked in either 5% BSA in TBST (for phospho-cPLA2) or 5% nonfat milk in TBST (for actin) and incubated overnight with Abs against phospho-cPLA2 (1:1000 in 5% BSA in TBST) and actin (1:10,000 in 5% milk in TBST). Secondary Abs used for detection were diluted 1:7500 in 5% BSA in TBST (goat anti-rabbit HRP) or in 5% milk in TBST (goat anti-mouse HRP).

### cPLA_2_ and PLA_1_ activity measurements

F4/80^+^ splenocytes were lysed after incubating them for the indicated times with 25 nM 16:4(n-3) [or 25 nM 16:4(n-3) and a 10-min preincubation of 10 μM AH-7614], and cPLA_2_ or PLA_1_ activity was measured by using EnzChek PLA_2_ or EnzChek PLA_1_ assay kit, respectively (Thermo Fisher Scientific), according to manufacturer protocol.

### Ca^2+^ mobilization assay

GPR120 and GPR40 Flp-In T-REx 293 cells were plated at 50,000 cells/well in black 96-well plates with clear bottoms. Cells were then treated with 100 ng/ml doxycycline to induce receptor expression and were maintained overnight at 37°C and 5% CO_2_ before use. Cells were labeled for 45 min with Fura2-AM followed by washing and maintenance in HBSS. Fura-2 fluorescent emission at 510 nm resulting from 340 or 380 nm of excitation was then monitored before and for 90 s after the addition of test compound using a Flexstation II plate reader (Molecular Devices, Sunnyvale, CA, USA). Ca^2+^ responses were taken as the peak 340/380 ratio measured after compound addition and normalized to the maximal response obtained to either α-linolenic acid (αLA) or 16:4(n-3) as indicated.

### β-Arrestin2 recruitment assay

HEK293T cells were cotransfected by using polyethyleneimine with FLAG-GPR120-eYFP and nanoluciferase (NLUC)-β-arrestin2 plasmids or FLAG-GPR40-eYFP and NLUC-β-arrestin2 plasmids in a ratio of 4:1. At 24 h post-transfection, cells were plated into poly-d-lysine–coated, white, 96-well tissue culture plates and maintained for a further 24 h before experiments. For experiments, cells were first washed and then incubated at 37°C for 30 min in HBSS. NLUC NanoGlo substrate (Promega, Madison, WI, USA) was added with a final dilution of 1:800 before a further incubation for 10 min at 37°C. Test compounds were then added at the specified concentration, and cells incubated for a final 5 min before measuring luminescent emission at 530 and 465 nm using a ClarioStar plate reader (BMG Labtech, Cary, NC, USA). The 530/465 emission ratio was calculated and corrected for the ratio obtained in cells that were transfected with only the NLUC-β-arrestin2 plasmid before being normalized to the maximal response obtained to 16:4(n-3).

### Patient samples

Blood samples were collected from 35 patients with cancer before and 4 h after receiving chemotherapy at the University Medical Center Utrecht. The study was approved by the Institutional Ethical Review Board of the University Medical Center Utrecht, and written informed consent was obtained from all patients. Blood was collected in a cell preparation tube. Peripheral blood mononuclear cells and plasma were isolated. Plasma was stored immediately at −80°C. Levels of LPC(24:1) were determined in the citrate plasma samples of 19 patients who received platinum-based chemotherapy and 16 patients who received non–platinum-based chemotherapy.

### 16:4(n-3) measurements

16:4(n-3) measurements were performed as previously described (21). In brief, a total lipids fraction was extracted from conditioned medium derived from cisplatin-stimulated murine bone marrow mesenchymal stem cells by a modified Bligh and Dyer extraction performed according to Retra *et al.* (28). Lipid extracts were analyzed by using single reaction monitoring on a triple-quadrupole mass spectrometer (Xevo, Waters, St. Quentin, France) supported by ultra-performance liquid chromatography (Waters).

### LPC analysis of sCM samples and patient plasma samples

Total lipids from sCM or plasma were extracted by using a modified Bligh and Dyer extraction performed according to Retra *et al.* (28). LPC analysis was performed by using a modified method of Retra *et al.* (28). Here, the HPLC column was replace by a fused core HALO C18 column (Biotech, Onsala, Sweden). To increase sensitivity, LPCs were detected in multiple reaction monitoring mode, in which a collection of 36 different LPC masses (14:0 to 26:6 LPC) were monitored and confirmed by the formation of a phosphocholine fragment of 184 *m*/*z* (collision energy was 55 V). Furthermore, the fatty acid composition of LPCs was confirmed by the presence of the corresponding fatty acid fragment in a second mass spectrometry analysis. To quantify LPC(24:1) levels in patient plasma, a standard curve of LPC(24:1) was used.

### LPC(24:1) production

1,2-Dinervonoyl-phosphatidylcholine [PC(24:1/24:1)] was purchased from Avanti Polar Lipids (chloroform dissolved; Alabaster, AL, USA) and converted *in vitro* into LPC(24:1) by addition of sPLA_2_. Chloroform was evaporated under a stream of nitrogen to yield 20 µmol of dried PC(24:1), after which it was sonicated in 1 ml PLA_2_ buffer (10 mM TRIS-HCl, 100 mM NaCl, 2 mM CaCl_2_, pH 7.4) that contained 1% lipid-free BSA to form liposomes. Incubation with 2 U of pancreas sPLA_2_ for 20 h at ambient temperature yielded an 80–90% conversion of PC(24:1/24:1) to LPC(24:1). The conversion was monitored by mass spectrometry. Subsequently, proteins (BSA and PLA_2_) were removed by a Bligh and Dyer lipid extraction.

### Statistical analysis

All data are presented as means ± sem. Statistical significance for all animal experiments in which tumor volumes were assessed was determined by 1-way ANOVA with Tukey’s correction for multiple testing. All other data were analyzed by using 2-tailed Student’s *t* test. A value of *P* < 0.05 was considered statistically significant. Levene’s test was used to determine whether variance between groups was comparable. Animals were excluded from analysis if ≥2 tumor measurements were significant outliners compared with the rest using the Grubbs outlier test (α = 0.05).

## RESULTS

### 16:4(n-3) is an agonist for both GPR40 and GPR120

**Figure 1.**
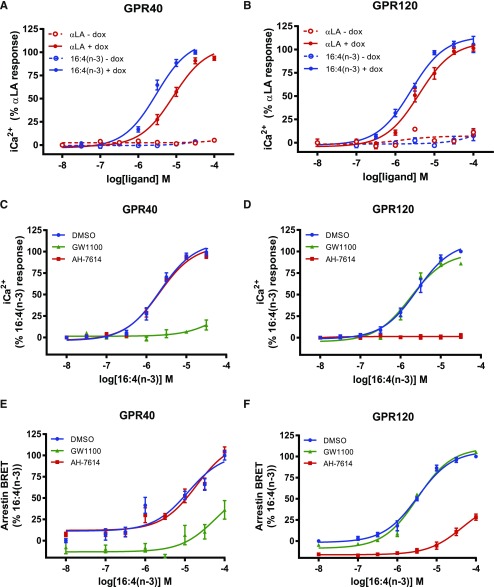
16:4(n-3) induces elevation of [Ca^2+^]_i_ and recruitment of β-arrestin2 in GPR40- and GPR120-expressing cells. *A*, *B*) 16:4(n-3) is able to induce a [Ca^2+^]_i_ response in GPR40- (*A*) and GPR120-expressing (*B*) Flp-In T-REx 293 cells. 16:4(n-3) activates both receptors with a higher potency than αLA, which was used as a positive control; Flp-In T-REx 293 cells without doxycycline (dox; *i.e.,* no GPR40/120 expression) were used as negative controls. *C*) A GPR40-specific antagonist (GW1100, 10 μM) was able to block 16:4(n-3)-mediated activation of GPR40, whereas a GPR120-specific antagonist (AH-7614, 10 μM) had no effect. *D*) AH-7614 was able to block 16:4(n-3)-mediated activation of GPR120, whereas GW1100 had no effect. *E*, *F*) β-Arrestin2 is recruited to both GPR40 (*E*) and GPR120 (*F*) receptors after stimulation with increasing concentrations of 16:4(n-3). Recruitment to GPR40 was blocked by addition of GW1100 but not by AH-7614. Recruitment of β-arrestin2 to GPR120 upon stimulation with 16:4(n-3) was inhibited by AH-7614 but not GW1100. Panels *A* and *B* show grouped results of 2 independent experiments with similar outcome, whereas panels *C*–*F* show grouped results of 3 independent experiments with similar outcome. BRET, bioluminescence resonance energy transfer. Data are presented as means ± sem.

**Figure 2.**
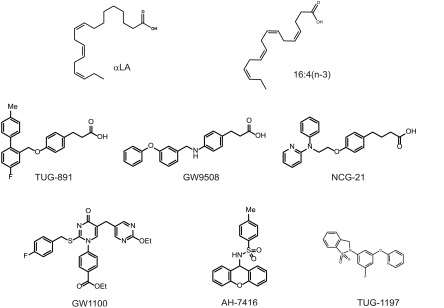
Chemical structures of GPR40 and/or GPR120 agonists and antagonists—all compounds used in this study.

To assess whether 16:4(n-3) might activate GPR40 and/or GPR120, we performed [Ca^2+^]_i_ flux experiments using an HEK293-based cell system that allowed inducible expression of either GPR40 or GPR120. 16:4(n-3) produced a concentration-dependent elevation of [Ca^2+^]_i_ in cells that expressed either GPR40 or GPR120, whereas this was absent from cells that were not induced to express these receptors (**Fig. 1*A*, *B***), which demonstrated that this fatty acid is an agonist of both GPR40 and GPR120. The potency of 16:4(n-3) to produce [Ca^2+^]_i_ responses *via* each GPCR was somewhat greater than the more widely studied 18:3(n-3) fatty acid, αLA, which was used as a positive control. The effect of 16:4(n-3) in GPR40-expressing cells was blocked by pretreatment with the GPR40-specific antagonist, GW1100 (3), but not by the GPR120-specific antagonist, AH-7614 (Fig. 1*C*) (26). By contrast, in GPR120-expressing cells, AH-7614 but not GW1100 prevented response to 16:4(n-3) (Fig. 1*D*). In addition, 16:4(n-3) also promoted recruitment of β-arrestin2 to both GPR40 and GPR120. This again was blocked at each receptor only by the corresponding antagonist (Fig. 1*E*, *F*). All compounds used in this study are listed in **Table 1** and their structures shown in **Fig. 2**. Taken together, 16:4(n-3) is a novel agonist of GPR40 and GPR120.

**TABLE 1.** GPR40 and GPR120 agonists and antagonists

Compound	pEC_50_ GPR40	pEC_50_ GPR120	Formula	Study
Agonist				
TUG-891	4.2	7.4	C_23_H_21_FO_3_	Shimpukade *et al*. (25), Hudson *et al*. (30)
GW9508	6.6–7.3	5.5	C_22_H_21_NO_3_	Briscoe *et al*. (3), Hudson *et al*. (30)
NCG21	4.7	5.9	C_23_H_23_N_2_O_3_	Sun *et al*. (24), Hudson *et al*. (30)
TUG-1197	<4.0	6.9	C_18_H_13_N_2_O_3_	Azevedo *et al*. (27)
Antagonist				
GW1100	6.0	<5.0	C_27_H_25_FN_4_O_4_S	Briscoe *et al*. (3)
AH-7614	<4.6	7.1	C_20_H_17_NO_3_S	Sparks *et al*. (26)

### GPR40 and GPR120 are expressed on splenic macrophages

**Figure 3.**
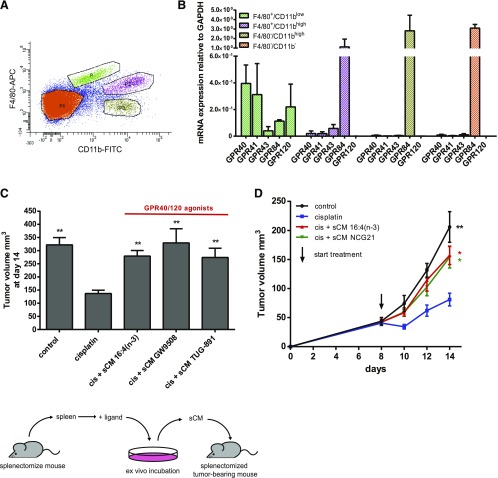
GPR40 and GPR120 are expressed by splenic macrophages, and dual GPR40 and GPR120 agonists recapitulate 16:4(n-3)-mediated chemoresistance *in vivo*. *A*) FACS analysis of BALB/c mouse spleen stained for F4/80 and CD11b. *B*) Indicated populations were sorted and relative expression of the free fatty acid receptors GPR40, GPR41, GPR43, GPR84, and GPR120 on these populations was determined by quantitative PCR. mRNA expression levels were normalized to GAPDH (glyceraldehyde 3-phosphate dehydrogenase) control. *C*) sCM derived from splenocytes that were incubated with the dual GPR40 and GPR120 agonists TUG-891 or GW9508 induce chemoresistance *in vivo* to an extent similar to sCM derived from splenic macrophages that were incubated with 16:4(n-3). The illustration below the data sets illustrates the approach used to generate sCM and its injection into tumor-bearing mice. *D*) Coadministration of cisplatin (cis) and sCM from splenocytes that were cultured in the presence of NCG21 (another GPR40/GPR120 agonist) induces chemotherapy resistance in splenectomized, tumor-bearing BALB/c mice. Panels *B* and *D* show grouped results of 2 independent experiments with similar outcomes (*B*: *n* = 4 per group; *D*: *n* = 8 per group). Panel *C* shows grouped results of 3 independent experiments with similar outcomes (*n* = 12 per group). Data are presented as means ± sem. Statistical significance was determined by 1-way ANOVA (*C*, *D*) or 2-tailed Student’s *t* test (*B*); all compared with cisplatin alone unless indicated otherwise. *P < 0.05; **P < 0.01.

To assess the relevance of these pharmacologic experiments to resistance-inducing splenic macrophages *in vivo*, we isolated splenic subpopulations on the basis of their expression of macrophage markers F4/80 and CD11b (**Fig. 3*A***). Quantitative RT-PCR analysis showed that GPR40 and GPR120 were expressed exclusively within the spleen by F4/80^+^/CD11b^low^ splenocytes (Fig. 3*B*) that have previously been shown to induce 16:4(n-3)-mediated chemoresistance (22). The F4/80^+^/CD11b^low^ population also expressed detectable levels of both short-chain fatty acid receptors, GPR43 and GPR41; however, these receptors respond only to fatty acids with a chain length of <7 carbons (29) and were not considered further.

### GPR40 and GPR120 agonists recapitulate 16:4(n-3)-mediated chemoresistance *in vivo*

We next assessed whether the synthetic dual GPR40 and GPR120 agonists, GW9508 and TUG-891, could induce chemotherapy resistance *in vivo*. Isolated splenocytes cultured *in vitro* were treated with fatty acid 16:4(n-3) or with either GW9508 or TUG-891. This sCM was then injected into splenectomized tumor-bearing mice, along with cisplatin. As observed previously (21, 22), the reduction in tumor volume that was produced 14 d after cisplatin treatment was abolished by coinjection of 16:4(n-3)–sCM. This effect was fully recapitulated by conditioned medium that was generated after treatment of splenocytes with either GW9508 or TUG-891, which implied that activation of GPR120 and/or GPR40 is either directly involved in, or can mimic, 16:4(n-3)-mediated chemoresistance *in vivo* (Fig. 3*C*). Although GW9508 is a GPR40-selective agonist (3), it is well established to also display agonism at GPR120 and, indeed, has often been used as an activator of GPR120 in cell types in which GPR40 is not expressed [*e.g.,* Oh *et al*. (9)]. Similarly, although TUG-891 is a GPR120-selective agonist, it also displays significant potency at GPR40, particularly at the mouse ortholog of this receptor (30). In addition, sCM that is derived from splenocytes incubated with NCG21, another GPR40 and GPR120 dual agonist (31), also induced chemoresistance (Fig. 3*D*).

### Antagonism of GPR120 prevents 16:4(n-3)-mediated chemoresistance *in vivo*

**Figure 4.**
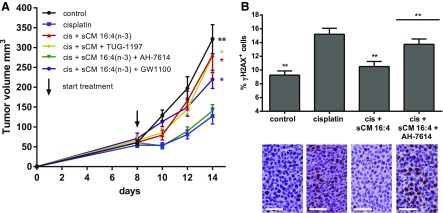
16:4(n-3)-induced chemotherapy resistance is mediated *via* GPR120 *in vivo*. Treatment of C26 tumor-bearing BALB/c mice with cisplatin (cis) and sCM derived from splenocytes that were stimulated with GPR120-specific agonist TUG-1197 induces chemoresistance, which indicates a central role for GPR120 in 16:4(n-3)-mediated resistance. *A*) GPR120-specific antagonist AH-7614 was able to completely block 16:4(n-3)-mediated resistance, whereas GPR40-specific antagonist GW1100 was ineffective. Tumors of mice that were treated with cisplatin and sCM + 16:4(n-3) showed a decrease in γH2AX levels 4 h after treatment compared with animals treated with cisplatin alone. *B*) Mice that were treated with cisplatin and sCM + 16:4(n-3) + AH-7614 had γH2AX levels similar to mcie treated with cisplatin alone. Scale bars, 50 μm. All experiments in this figure were conducted in C26 tumor-bearing BALB/c mice. All graphs show grouped results of 2 independent experiments with similar outcomes (*n* = 8 per group). Data are presented as mean ± sem. Statistical significance was determined by 1-way ANOVA (*A*) or 2-tailed Student’s *t* test (*B*); all compared with cisplatin alone unless indicated otherwise. *P < 0.05; **P < 0.01.

Because F4/80^+^/CD11b^low^ splenocytes express both GPR40 and GPR120, and GW9508, TUG-891, and NCG21 are not selective enough to differentiate between the 2 receptors in this system, we used additional pharmacologic tool compounds to determine which receptor(s) were relevant for 16:4(n-3)-mediated chemoresistance. First, we tested the ability of a GPR120-specific antagonist—AH-7614—and a GPR40-specific antagonist—GW1100—to block the chemoresistance effect of 16:4(n-3) *in vivo*. We found that preincubation of splenic macrophages with AH-7614 (GPR120 antagonist) completely prevented 16:4(n-3)-mediated chemoresistance, whereas this was not achieved with the GPR40-specific antagonist GW1100 (**Fig. 4*A***). These results implied that 16:4(n-3) produces chemoresistance by activating GPR120 and not GPR40. To further support this conclusion, we tested the ability of a recently described GPR120-specific agonist—TUG-1197—that has no activity at GPR40 (27) to induce chemoresistance. As anticipated, coadministration of cisplatin and conditioned medium derived from splenocytes that were incubated with TUG-1197 induced chemoresistance to an extent similar to sCM from 16:4(n-3)-exposed splenocytes (Fig. 4*A*).

PIFAs are able to alter the DNA damage response in tumor cells, which leads to chemoresistance. Compared with cisplatin monotherapy, coadministration of 16:4(n-3)–sCM with cisplatin led to a decrease in γH2AX levels—a measure of double strand DNA breaks—4 h after treatment. By contrast, in mice that were treated with conditioned medium from splenocytes that were cotreated with 16:4(n-3) and AH-7614, this effect was absent (Fig. 4*B*). Antagonism of GPR120 thus blocks chemoresistance both in tumor volumetric experiments and at the level of γH2AX in tumors, which indicates that GPR120 is the functional receptor transducing 16:4(n-3)-mediated chemoresistance.

### sCM derived from GPR120^−/−^ mice fails to induce 16:4(n-3)-mediated chemoresistance

**Figure 5.**
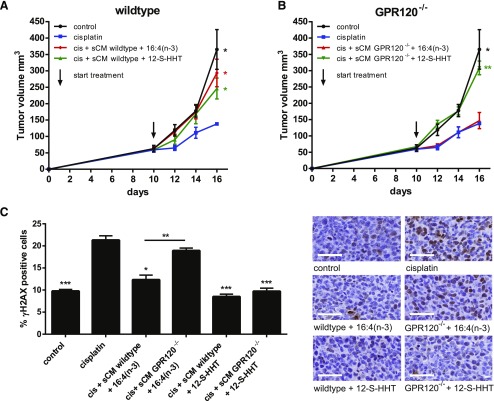
sCM derived from GPR120^−/−^ mice is unable to induce 16:4(n-3)-mediated chemoresistance. *A*) sCM from wild-type C57BL/6 mice that were incubated with either 16:4(n-3) or 12-S-HHT was able to induce resistance against cisplatin (cis) in wild-type C57BL/6 splenectomized, tumor-bearing mice. *B*) sCM of GPR120^−/−^ mice that were incubated with 16:4(n-3) was unable to induce chemoresistance when coinjected with cisplatin in wild-type LLC tumor-bearing C57BL/6 mice, whereas sCM from GPR120^−/−^ mice that were incubated with 12-S-HHT retained the ability to induce resistance. *C*) γH2AX levels in tumors treated with cisplatin and sCM GPR120^−/−^ + 16:4(n-3) were similar to those of tumors treated with cisplatin monotherapy, whereas tumors that were treated with cisplatin and sCM wild-type + 16:4(n-3) or sCM wild-type + 12-S-HHT or sCM GPR120^−/−^ + 12-S-HHT showed significantly lower γH2AX levels. Scale bars, 50 μm. All graphs show grouped results of 2 independent experiments with similar outcomes (*n* = 8 per group). Data are presented as means ± sem. Statistical significance was determined by 1-way ANOVA (*A*, *B*) or 2-tailed Student’s *t* test (*C*); all compared with cisplatin alone unless indicated otherwise. *P < 0.05; **P < 0.01; ***P < 0.001.

Pharmacologic characterization suggested that the effect of 16:4(n-3) is produced *via* activation of splenocyte-expressed GPR120; therefore, we hypothesized that the chemoresistance effect of 16:4(n-3) would be absent if splenocytes from GPR120-deficient mice were used. As anticipated, we found that sCM obtained from splenocytes that were isolated from GPR120^−/−^ mice and incubated with 16:4(n-3) did not induce chemoresistance when introduced into wild-type, tumor-bearing mice, whereas sCM from wild-type mice on the same genetic background did (**Fig. 5*A*, *B***). This was true whether chemoresistance was assessed *via* tumor volumetric measurements or γH2AX immunohistochemical analysis (Fig. 5*C*).

16:4(n-3) is not the only PIFA (21). The hydroxyl fatty acid, 12-*S*-hydroxy-5,8,10-heptadecatrienoic acid (12-S-HHT), is known to induce chemoresistance by activating leukotriene B4 receptor signaling in splenic macrophages. As anticipated, the capacity of 12-*S*-HHT to cause chemoresistance was not hampered by the genetic loss of GPR120 (Fig. 5*B*, *C*).

### Activation of GPR120 by 16:4(n-3) leads to enhanced cPLA_2_ activity

**Figure 6.**
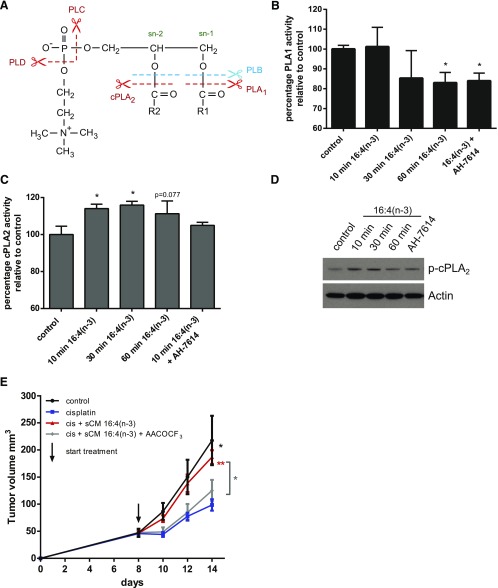
16:4(n-3) stimulation increases cPLA_2_ activity in splenic macrophages. *A*) Schematic overview of hydrolysis sites of PC to yield metabolites, such as LPC. Splenic macrophages were harvested from murine BALB/c spleens by magnetic bead sorting using F4/80 Abs. Macrophages were incubated with either vehicle or 16:4(n-3) (25 nM) for indicated times. *B*, *C*) Fluorescent-based analysis of PLA_1_ and cPLA_2_ enzymatic activity revealed a decrease in PLA_1_ activity (*B*) and a significant time-dependent increase in cPLA_2_ activity (*C*) upon stimulation with 16:4(n-3). Preincubation of splenic macrophages with the GPR120-specific antagonist AH-7614 did not affect 16:4(n-3)-mediated changes in PLA_1_ activity but was able to prevent the 16:4(n-3)-induced increase in cPLA_2_ activity. *D*) In addition, splenic macrophage cell lysates were analyzed by Western blot for phospho-cPLA_2_. Actin was used as a loading control. *E*) Treatment of tumor-bearing BALB/c mice with cisplatin (cis) and sCM derived from splenocytes that were incubated with 16:4(n-3) and AACOCF_3_ did not induce chemoresistance, whereas cotreatment with cisplatin and sCM+ 16:4(*n*-3) did. Panel *B* shows grouped results of 2 independent experiments with similar outcomes. Panels *C* and *D* show results of 4 independent experiments. Panel *E* shows grouped results of 2 independent experiments with similar outcomes (*n* = 8 per group). Data are presented as means ± sem. Statistical significance was determined by 2-tailed Student’s *t* test (*B*, *C*) or 1-way ANOVA (*E*); all compared with vehicle control (*B*, *C*) or with cisplatin alone (*E*) unless indicated otherwise. *P < 0.05; **P < 0.01.

Having established a role for GPR120 that is expressed by F4/80^+^/CD11b^low^ splenocytes in initiation of resistance to cisplatin, we next set out to define the mechanistic basis for this effect. It is known that PIFA-activated splenic macrophages produce 6 unsaturated LPCs that have the potential to act as effector molecules of resistance to cisplatin (22). Production of LPC from PC may be mediated by various enzymes, which leads to the generation of structurally different LPCs. PLA_1_ can hydrolyze PC at the sn-1 position and cPLA_2_ can hydrolyze PC at the sn-2 position, whereas PLB can cleave PC at both positions (**Fig. 6*A***). To investigate which of these enzymes was involved in 16:4(n-3)-mediated LPC production, we examined PLA_1_ and cPLA_2_ activity in splenic macrophages. We found no up-regulation of PLA_1_ activity in splenic macrophages upon treatment with 16:4(n-3) (Fig. 6*B*). Moreover, although a statistically significant decrease in PLA_1_ activity was detected after sustained exposure to 16:4(n-3), this did not reflect activation of GPR120 because the effect was not prevented by coincubation with the GPR120-specific antagonist, AH-7614 (Fig. 6*B*). We noted a significant increase in cPLA_2_ activity in the splenic macrophage population after exposure to 16:4(n-3) *via* both immunoblotting to detect active, phosphorylated cPLA_2_ (Fig. 6*C*) and by directly measuring the enzymatic activity of cPLA_2_ (Fig. 6*D*). This effect was blocked by pretreatment of splenocytes with GPR120 antagonist AH-7614 (Fig. 6*C*, *D*). To link cPLA_2_ activity in splenocytes to the chemoresistance effect of 16:4(n-3), we injected sCM generated from splenocytes that were incubated with 16:4(n-3) and the cPLA_2_ inhibitor, AACOCF_3_, into tumor-bearing mice and observed that the chemoresistance effect of 16:4(n-3) was abolished by the cPLA_2_ inhibitor (Fig. 6*E*).

### GPR120 mediates 16:4(n-3) chemoresistance *via* production of LPC(24:1)

**Figure 7.**
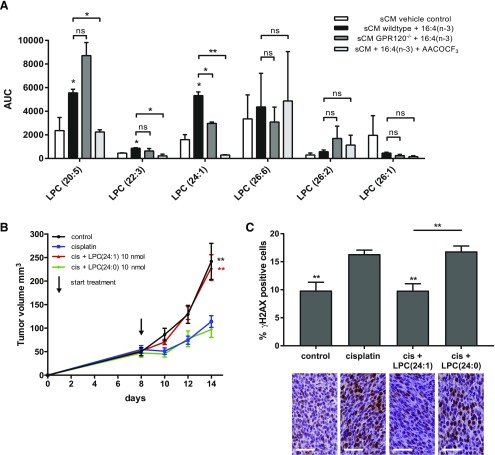
LPC(24:1) secretion is specifically up-regulated upon 16:4(n-3) stimulation of splenic macrophages. *A*) LPC analysis of sCM from wild-type mice that were incubated with 16:4(n-3) revealed 3 significantly up-regulated unsaturated LPCs compared with vehicle control–incubated splenic macrophages. LPC(24:1) up-regulation in wild-type sCM was lost in GPR120^−/−^ sCM. In addition, LPC(24:1) was also lost when splenocytes were incubated with 16:4(n-3) in the presence of the PLA_2_ inhibitor AACOCF_3_. *B*) Coadministration of cisplatin (cis) and 10 nmol LPC(24:1) was sufficient to induce chemotherapy resistance, whereas coadministration of cisplatin and 10 nmol of LPC(24:0) could not. *C*) Tumors of mice that were treated with cisplatin and LPC(24:1) had levels of γH2AX similar to control mice, whereas mice that were treated with cisplatin alone or cisplatin and LPC(24:0) showed increased levels of γH2AX compared with control mice. Analysis of γH2AX was performed 4 h post-treatment. Scale bars, 50 μm. Experiments in Panels *B* and *C* were conducted in C26 tumor-bearing BALB/c mice. All graphs show combined results of 2 independent experiments with similar outcomes (*A*: *n* = 4 per group; *B*: *n* = 8 per group; *C*: *n* = 8 per group). AUC, area under the curve; ns, not significant. Data are presented as means ± sem. Statistical significance was determined by 1-way ANOVA (*B*, *C*) or 2-tailed Student’s *t* test (*A*, *D*); all compared to cisplatin alone unless indicated otherwise. *P < 0.05; **P < 0.01.

PIFA-activated splenic macrophages that induce chemoresistance produce 6 chemically distinct, unsaturated LPCs. By using a lipidomics approach, we identified which of these LPCs was released specifically upon 16:4(n-3) stimulation of splenocytes from wild-type mice and whether this was altered in splenocytes that were isolated from GPR120^−/−^ animals. When measuring LPC levels in sCM derived from wild-type splenocytes that were incubated with 16:4(n-3), we found a significant up-regulation of 3 these 6 LPCs, namely LPC(20:5), LPC(22:3), and LPC(24:1) (**Fig. 7*A***). By contrast, when equivalently conditioned medium from splenocytes that were isolated from GPR120^−/−^ animals was analyzed, only LPC(20:5) and LPC(22:3) were up-regulated, which suggested a specific role for GPR120 in the release of LPC(24:1) (Fig. 7*A*). Of importance, the cPLA_2_ inhibitor, AACOCF_3_, also greatly reduced LPC(24:1) production from splenocytes in response to 16:4(n-3) treatment (Fig. 7*A*). To test directly if LPC(24:1) was the resistance-inducing molecule secreted by 16:4(n-3)-activated splenic macrophages, we coinjected LPC(24:1) with cisplatin into splenectomized, tumor-bearing mice and found that this treatment did indeed induce chemoresistance, whereas the closely related LPC(24:0) did not (Fig. 7*B*). In line with these findings, immunohistochemical analysis of tumors that were treated with cisplatin and LPC(24:1) showed decreased levels of γH2AX^+^ cells compared with cisplatin monotherapy or cisplatin combined with LPC(24:0) (Fig. 7*C*).

### GPR120-induced chemoresistance by 16:4(n-3) is context dependent

We next assessed whether our findings could be generalized to other macrophage-like populations. RAW264.7 cells are a macrophage-like cell line known to express GPR120. In such cells, activation of GPR120 is able to suppress production of inflammatory mediators, including TNF-α, that are induced by treatment with such agents as LPS (30). In addition, it has been shown that activation of cPLA_2_ *via* GPR120 in RAW264.7 cells induces an anti-inflammatory response (10); however, sCM derived from RAW264.7 cells that were stimulated with 16:4(n-3) did not induce chemoresistance, nor did we find an up-regulation of phospho-cPLA2 in, or production of, LPC(24:1) by these cells, which indicated that GPR120-induced production of 16:4(n-3)-mediated chemoresistance is context dependent (Supplemental Fig. 2).

### Human splenic macrophages express GPR120, and LPC(24:1) is detected in patients who received platinum-based chemotherapy

**Figure 8.**
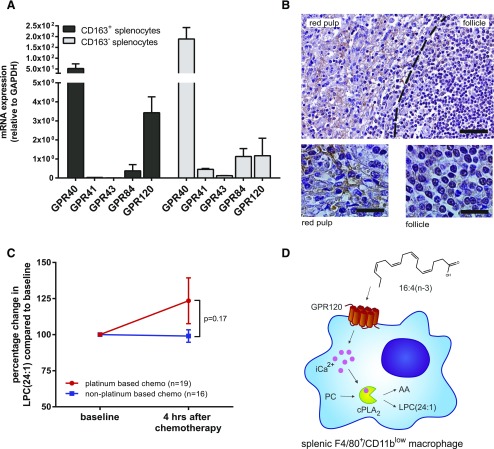
Human splenic macrophages express GPR120, and LPC(24:1) is increased in blood plasma of patients who were treated with platinum-containing chemotherapeutics. *A*) Relative expression of GPR40, GPR41, GPR43, GPR84, and GPR120 in human CD163^+^ and CD163^−^ splenocytes was determined by quantitative PCR. GPR40 and GPR120 are expressed by human splenocytes. *B*) Immunohistochemical staining of GPR120 on human splenic tissue reveals GPR120^+^ cells in red pulp of the spleen. Scale bars, 100 μm (top), 20 μm (bottom). *C*) LPC(24:1) measurements in patient plasma showed increased LPC(24:1) levels in patients after treatment with platinum-based chemotherapeutics but not in patients who were treated with non–platinum-based chemotherapeutics (*n* = 19 platinum-based chemotherapy; *n* = 16 non–platinum-based chemotherapy). *D*) Overview illustration of the identified 16:4(n-3)–GPR120 signaling axis in splenic macrophages. Panel *A* shows representative results of 2 independent experiments. GAPDH, glyceraldehyde 3-phosphate dehydrogenase. Data are presented as means ± sem. Statistical significance was determined by 2-tailed Student’s *t* test.

To assess the potential clinical applicability of our chemoresistance model, we determined expression levels of GPR40, GPR41, GPR43, GPR84, and GPR120 in human CD163^+^ and CD163^−^ splenocytes. CD163 is a marker for splenic red pulp macrophages in humans and is the closest analog to murine F4/80. Human CD163^+^ splenocytes showed increased expression of GPR40 and GPR120 compared with CD163^−^ splenocytes, which correlated with findings in mice; however, some GPR120 was also detected in CD163^−^ splenocytes, which was indicative of a broader expression pattern in humans compared with mice (**Fig. 8*A***). In support of this, immunohistochemistry showed stronger GPR120 staining in red pulp of the spleen (Fig. 8*B*). The splenic red pulp harbors tissue resident macrophages that are known for their role in iron homeostasis and has previously been identified as the location for murine F4/80^+^/CD11b^low^ macrophages that are known to induce chemoresistance (22, 32). Next, we measured LPC(24:1) levels in patients who were treated with either platinum-containing chemotherapeutics or non–platinum-containing chemotherapeutics before and 4 h after administration of chemotherapy. We found increased plasma LPC(24:1) levels 4 h after treatment in patients who received platinum-based chemotherapy compared with patients who were treated with non–platinum-based chemotherapeutics (Fig. 8*C*). Additional studies will reveal if LPC(24:1) can be used as an early marker for chemotherapy resistance in platinum-treated patients.

## DISCUSSION

Here, we identified the PIFA, 16:4(*n*-3), as a novel and endogenously produced agonist of GPR120 and describe a function for GPR120 in inducing chemotherapy resistance. 16:4(n-3)–GPR120 signaling in splenic macrophages enhances production and release of LPC(24:1), thus leading to chemotherapy resistance *in vivo* (Fig. 8*D*). GPR120 is a receptor for long-chain fatty acids (2) and, in certain studies, activation by ω-3 fatty acids of GPR120 has been highlighted (5, 9, 10). The relatively high expression of GPR120 in adipose tissue, macrophages, and intestine has resulted, to date, in a focus on its contribution to insulin signaling, obesity, and anti-inflammatory responses and, on the basis of these, the potential for GPR120 agonists to regulate glucose homeostasis and act as potential medicines to treat type II diabetes (7). Various studies, however, have also suggested a role for GPR120 in tumor progression. Overexpression of GPR120 in colorectal carcinomas has been associated with angiogenic switching and cell motility (33). Similarly, in pancreatic cancer cell lines, down-regulation of GPR120 prevented cell migration (34). In contrast, in prostate cancer, GPR120 signaling seems to have an antitumor effect (35).

Numerous distinct fatty acids can activate GPR120, and their downstream effects may differ between cell and tissue types, generating the reported complexity of fatty acid signaling in cancer biology. Here, we identified 16:4(n-3) as a novel agonist of GPR120 and showed that activation of GPR120 by 16:4(n-3) in splenic macrophages leads to increased cPLA_2_ activity and the associated increased secretion of LPC(24:1), which subsequently results in resistance to chemotherapy *in vivo*. Of interest, measurements of LPC(24:1) levels in the plasma of patients who received platinum-based chemotherapy showed an increase 4 h after administration of chemotherapy compared with baseline levels, which is suggestive of a human correlate with mouse-based studies.

It is important to note, however, that the capacity of GPR120 to initiate a signaling cascade that results in secretion of LPC(24:1) is context dependent. Although macrophage-like model cell systems, such as RAW264.7 cells, are frequently used to explore aspects of macrophage biology and have been used to explore potential pathways linked to anti-inflammatory signals induced by GPR120 (10, 30), we were unable to observe production of LPC(24:1) upon addition of 16:4(n-3) to such cells. This demonstrates that such transformed cell lines may not always accurately mimic the behavior and function of primary cells.

That such transformed cell lines do not accurately mimic the behaviors and functions of native cells is perhaps intrinsically obvious, but these observations provide a useful cautionary note for potential overinterpretation of results that rely exclusively on such models.

Little is known about the function of LPCs, and, in particular, LPC(24:1), in tumor progression and anticancer drug resistance. Schneider *et al.* (36) showed that LPC and lysophosphatidic acid (LPA) induce migration and metastasis of rhabdomyosarcoma *in vitro* and *in vivo*. In addition, LPC and LPA levels increased when mice were treated with either vincristine or radiotherapy, postulating the hypothesis that the treatment-induced increase in LPC and LPA would facilitate metastasis. In contrast, Raynor *et al.* (37) showed that LPCs are rapidly metabolized by cancer cells and that they can prevent metastasis.

Because chemotherapy resistance poses one of the main problems in effective treatment of patients with cancer, insights into these mechanisms are of importance to identify new targets that could enhance the efficacy of chemotherapy. Our findings suggest that intricate lipid-signaling between noncancerous host cells and tumor cells can affect chemotherapeutic drug sensitivity. Identification of GPR120 as a key receptor in this process shows potential for the use of GPR120 antagonists to enhance chemotherapeutic efficacy.

## Supplementary Material

Supplemental Data

## Abbreviations

12-S-HHT: 12-*S*-hydroxy-5,8,10-heptadecatrienoic acid
16:4(n-3): hexadeca-4,7,10,13-tetraenoic acid
AACOCF_3_: arachidonyl trifluoromethyl ketone
BSA: bovine serum albumin
eYFP: enhanced yellow fluorescent protein
FACS: fluorescence-activated cell sorting
H2AX: histone 2A family member X
HRP: horseradish peroxidase
LPA: lysophosphatidic acid
LPC: lysophosphatidylcholine
αLA: α-linolenic acid
NLUC: nanoluciferase
PC: phosphatidylcholine
sCM: splenic conditioned medium
PIFA: platinum-induced fatty acid
TBST: Tris-buffered saline/Tween 20
