# Supplementary material for: Fatty acid 16:4(n-3) stimulates a GPR120-induced signaling cascade in splenic macrophages to promote chemotherapy resistance
Source: FASEB J. 2017 Feb 9;31(5):2195–209. doi: 10.1096/fj.201601248R (PMC5388545; doi:10.1096/fj.201601248R)
Supplement: Supplemental Data [file supp_31_5_2195__index.html]

Fatty acid 16:4(n-3) stimulates a GPR120-induced signaling cascade in splenic macrophages to promote chemotherapy resistance — Fatty acid 16:4(n-3) stimulates a GPR120-induced signaling cascade in splenic macrophages to promote chemotherapy resistance — Supplemental Data 

# Fatty acid 16:4(n-3) stimulates a GPR120-induced signaling cascade in splenic macrophages to promote chemotherapy resistance

## Supplemental Data

- Supplemental Data
