# Supplementary material for: Fatty acid 16:4(n-3) stimulates a GPR120-induced signaling cascade in splenic macrophages to promote chemotherapy resistance
Source: FASEB J. 2017 Feb 9;31(5):2195–209. doi: 10.1096/fj.201601248R (PMC5388545; doi:10.1096/fj.201601248R)
Supplement: Supplemental Data [file supp_fj.201601248R_Supplemental_Figures.pdf]

Supplemental figure 1

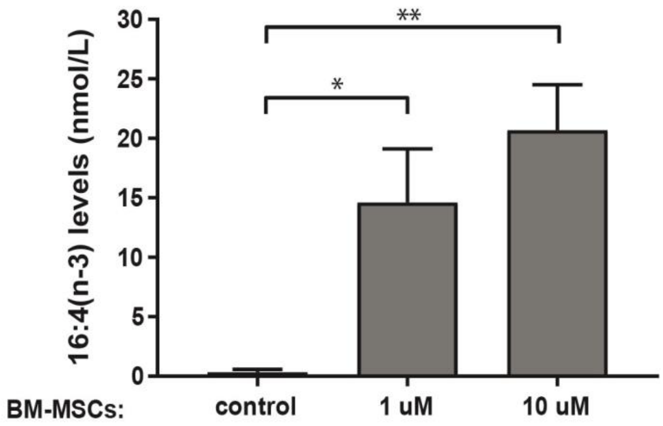

Supplemental figure 1: Bone marrow derived mesenchymal stem cells (BM-MSCs) produce 16:4(n-3) in response to cisplatin stimulation. Cultured MSCs were stimulated with vehicle, 1 uM cisplatin or 10 uM cisplatin for 4 hours. After 4 hours the medium was replaced by serum-free medium and harvested 24 hours later. This conditioned medium was analyzed by mass spectrometry for the presence of 16:4(n-3). Data shows combined data of two individual experiments with similar outcome. Data are expressed as mean ± SD.

Supplemental figure 2

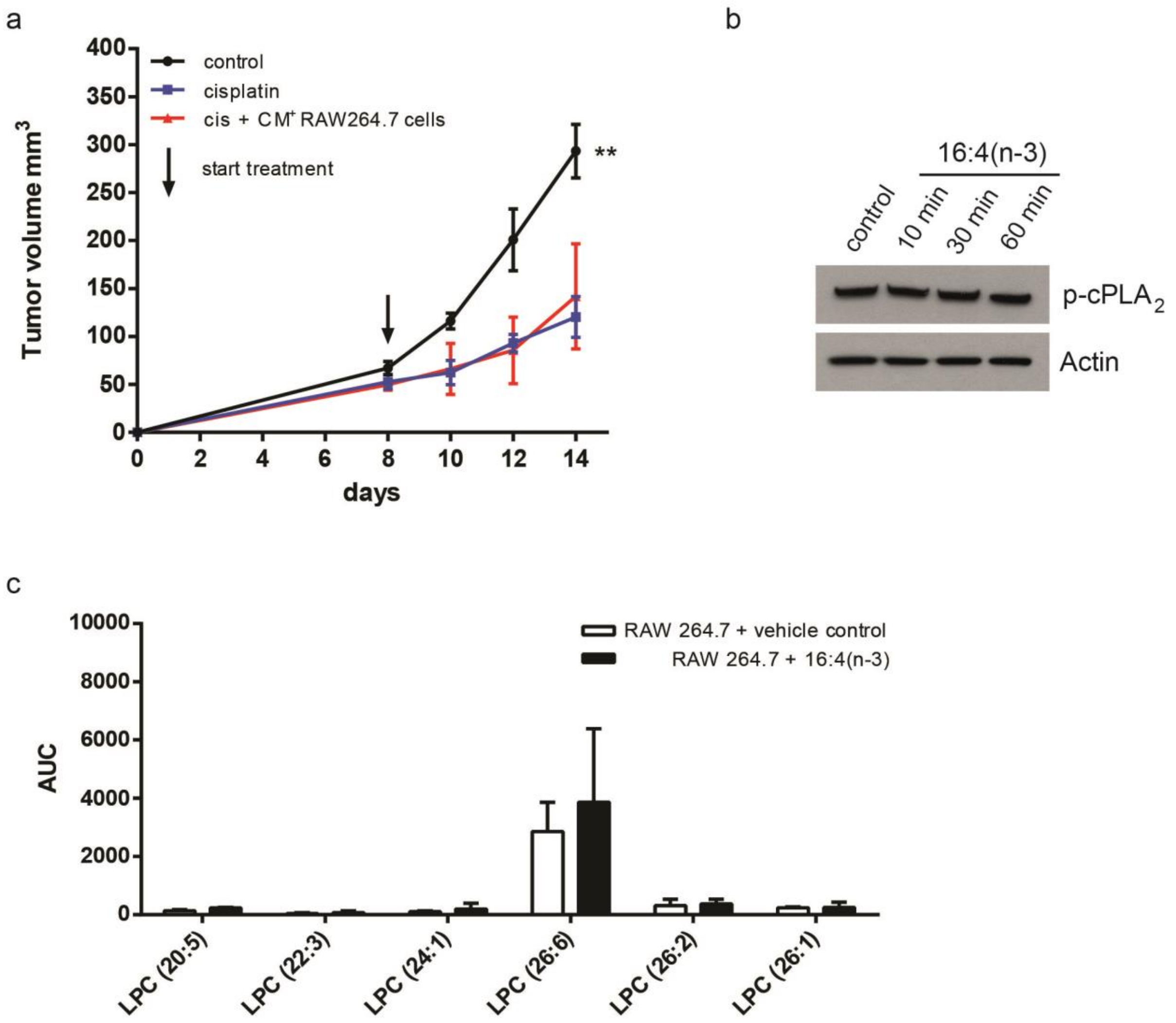

Supplemental figure 2: RAW264.7 cells do not induce chemotherapy resistance in response to 16:4(n-3). CM prepared from RAW264.7 cells stimulated with 16:4(n-3) does not induce chemoresistance in BALB/c mice (panel a). No increase in phospho-cPLA2 was seen by western blot analysis of RAW264.7 cells stimulated with 16:4(n-3) (25 nM) for indicated time points (panel b). LPC measurements in the CM from RAW264.7 cells incubated with either vehicle control or 16:4(n-3) showed overall low LPC levels and no significant changes between control and 16:4(n-3) stimulation (panel c). Graphs show results of two independent experiments (panel a n=8 per group, panel c 2 replicates per treatment). Data are represented as mean ± SEM. Statistical significance was determined by two-tailed Students T-test (panel c) or one-way ANOVA (panel a). Panel a: all compared to cisplatin alone * $P<0.05$ ** $P<0.01$.
